# Socioeconomic inequalities and COVID-19 – A review of the current international literature

**DOI:** 10.25646/7059

**Published:** 2020-10-09

**Authors:** Benjamin Wachtler, Niels Michalski, Enno Nowossadeck, Michaela Diercke, Morten Wahrendorf, Claudia Santos-Hövener, Thomas Lampert, Jens Hoebel

**Affiliations:** 1 Robert Koch Institute, Berlin Department of Epidemiology and Health Monitoring; 2 Robert Koch Institute, Berlin Department of Infectious Disease Epidemiology; 3 University of Düsseldorf Medical Faculty, Institute of Medical Sociology, Centre for Health and Society

**Keywords:** COVID-19, SARS-COV-2, HEALTH INEQUALITY, LITERATURE REVIEW

## Abstract

Social epidemiological research describes correlations between socioeconomic status and the population’s risk to become diseased or die. Little research of such correlations for SARS-CoV-2 and COVID-19 has so far been conducted. This scoping review provides an overview of the international research literature. Out of the 138 publications found, 46 were later included in the analysis. For the US and the UK, the reported findings indicate the presence of socioeconomic inequalities in infection risks as well as the severity of the course of the disease, with socioeconomically less privileged populations being hit harder. There are far fewer findings for Germany to date, as is the case for most other European countries. However, the scant evidence available so far already indicates that social inequalities are a factor in COVID-19. Most of these analyses have been ecological studies with only few studies considering socioeconomic inequalities at the individual level. Such studies at the individual level are particularly desirable as they could help to increase our understanding of the underlying pathways that lead to the development of inequalities in infection risks and the severity of disease and thereby could provide a basis to counteract the further exacerbation of health inequalities.

## 1. Introduction

Social epidemiological research from past decades has documented that the risk of becoming diseased or die is closely tied to the socioeconomic status [1, 2]. For a great number of diseases and causes of death, it has been shown that even in affluent countries with modern welfare systems these risks are distributed unequally in society. Becoming sick or dying prematurely is a risk that increases the lower a person’s socioeconomic status is [3–6]. Such health inequalities have been described in particular for chronic diseases and chronic infectious diseases such as tuberculosis [7]. But findings also indicate differences in frequency and severity for acute infections, such as viral respiratory diseases. Analyses of the 1918/1919 and 2009 influenza pandemics show that socioeconomically less privileged populations ran a greater risk of contracting and dying of influenza than socioeconomically more affluent groups [8–10]. However, such social epidemiological patterns can vary and change over the course of an epidemic, geographically and over time [11].

The SARS-CoV-2 (Severe Acute Respiratory Syndrome Coronavirus 2) virus, which was first identified in Wuhan, China, has caused a pandemic and led to historic challenges for societies around the world. It soon became clear that older people and those with pre-existing conditions in particular had a greater risk for a severe course of the coronavirus disease 2019 (COVID 19). But very little is so far known about the further social epidemiological patterns, although first reports from the UK and the US do point towards socioeconomic inequalities in COVID-19 [12, 13].

Socioeconomic inequalities in COVID-19 could result from unequal risks of becoming infected, that might, in turn, be caused by differences in living and working conditions [14]. Even during the pandemic, many people were living and working in conditions where they could scarcely protect themselves from an infection [13, 14]. One example is crowded living conditions, which can increase the risk of virus transmission. Data from the US showed that a severe course of an influenza infection was more frequent among people who lived in crowded households [15]. Similar findings for acute respiratory diseases were also reported from other countries [16, 17]. For COVID-19 as well, an increased risk of infection has been related to crowded living conditions [18, 19]. In many countries, in particular in urban settings, space for living is unequally distributed, with low income earners generally having less space, a finding applicable also to large German cities [20]. Socially disadvantaged populations living in shared accommodations (e.g. shelters) are as well regarded to be at higher risk for infection [21]. Working conditions can also be linked to differing infection risks. Key workers such as nurses, or those working in the logistics sector, retail and public transport, continued to work even during the pandemic and are generally in the middle to low income groups [22]. Working from home, a recommended measure to reduce infection risks, is an option open mainly to people on higher salaries and with higher qualifications [23].

Beyond these differences in the risk of becoming infected with SARS-CoV-2, certain risk factors for a severe course of COVID-19 are unequally distributed across different socioeconomic groups. These risk factors include cardiovascular diseases (for example coronary heart disease and hypertension), lung diseases (such as chronic bronchitis), liver diseases and diabetes. Cancer patients and patients with a compromised immune system are also considered COVID-19 risk groups [24]. Previous findings indicate that many of these conditions, for example coronary heart disease, diabetes, chronic bronchitis and, among men, also lung cancer, are more frequent in socioeconomically disadvantaged populations in Germany [25–28]. Smoking and obesity are currently discussed as two additional risk factors for a severe course of COVID-19 [29, 30] – risk factors also more present in socioeconomically disadvantaged groups [31, 32]. Psychosocial factors might as well influence whether and how severely a person develops an acute respiratory disease after being exposed to a virus [33]. An experimental study, for example, revealed that after being exposed to rhino and influenza viruses, people who describe themselves as being socially disadvantaged have a greater risk of developing acute symptoms of a upper respiratory tract infection [34]. Due to this socially unequal distribution of structural, health, behaviour related and psychosocial risk factors, it is plausible to assume that socioeconomically disadvantaged populations might be more susceptible to SARS-CoV-2 and at higher risk of a severe course of COVID-19.

This scoping review was conducted to answer the question if the international research literature provides any indications of socioeconomic inequalities in SARS-CoV-2 infection risk, severe courses of COVID-19 and increased mortality risk, and which social epidemiological patterns have been described so far.

## 2. Methodology

This literature review aims to provide a systematic overview of the international research literature on socioeconomic inequalities in COVID-19. As this is a first overview for a new and relatively broad research field, we followed the methodology of a scoping review [35, 36]. However, the number of databases searched was limited and both study selection and data extraction were conducted by a single person [37].

Peer-reviewed publications were identified through a search in the Pubmed database via the curated literature hub LitCovid. Not peer-reviewed publications were identified through searches in two large preprint servers. All available articles in the National Institute of Health’s (NIH) LitCovid database, currently the most complete resource for peer-reviewed publications on COVID-19 [38], as well as all publications up to 15 June 2020 on the medRxiv and bioRxiv preprint servers were screened. Only studies from countries with developed economies in Europe and North America, as listed by the United Nations [39], were included which reported epidemiological data on COVID-19 related to individual or regional-level socioeconomic variables (such as education, medium income, regional deprivation indices) in English or German language. Studies were excluded if they had an exclusive focus on ethnicity, did not consider individual or regional level socioeconomic indicators, were published after 15 June 2020 or did not report an own analysis of data. In addition, repeated searches (until 15 June 2020) were conducted in German and English in Google (for terms such as social, socioeconomic inequalities, education, profession, income and COVID-19 or SARS-CoV-2 or coronavirus) to identify ‘grey’ literature. Only publications by official bodies such as statistical offices or public health institutes, but also from foundations, private research institutions and non-governmental organisations were considered if they reported their own analyses of data. If they met the aforementioned criteria, the sources identified in newspaper articles were also included. Data extraction was based on a priori developed extraction chart and the results are presented based on the principles of a scoping review, i.e. without systematically assessing the quality of the evidence provided.

## 3. Results

**Figure 1:**
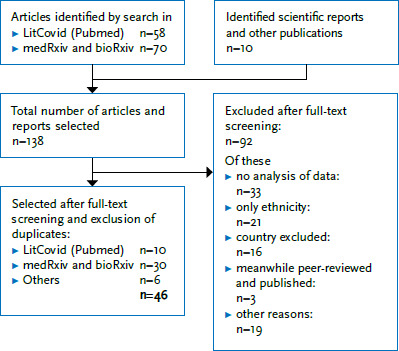
Flow diagram of inclusions and exclusions after title and abstract screening and the elimination of duplicates Source: Own diagram

For the period up to 15 June 2020, 5,248 articles on preprint servers (medRxiv n=4,225; bioRxiv n=1,023) and 22,306 articles from the LitCovid database were screened. After titles and abstracts were screened and the eligibility of the full texts was assessed, ten published and peer-reviewed articles and 30 not peer-reviewed publications were selected (Figure 1). Additional searches in Google and manual searches of the references yielded ten further publications, out of which six articles were finally included. Our literature review is therefore based on 46 publications (an overview of these publications is available online). 28 of these publications report findings from the US, 16 from the UK, one from Italy and one from Germany.

The articles included apply a highly heterogeneous group of measures of socioeconomic status. The majority (n=44) uses a set of regional level indicators (Table 1). Individual and regional level indicators are used by six studies, with only two using exclusively individual level variables.

Incidence and prevalence of SARS-CoV-2 infections, as well as hospitalisations, admissions to intensive care units or death linked to COVID-19 are the outcomes analysed by this study. Most studies (n=44) report socioeconomic inequalities with those in low socioeconomic status groups more affected. Seven studies also report inequalities for certain aspects where higher status groups are hit harder. Three studies find no correlations between the variables considered. A greater impact exclusively for socioeconomically more affluent groups is found in two not peer-reviewed studies [45, 62]. At the level of the correlations studied, twelve out of 74 (16%) regional level correlations found socioeconomic inequalities with more affluent groups being affected more and five out of 74 (7%) regional level correlations found no socioeconomic inequalities. Nine studies analysed a total of twelve correlations based on individual socioeconomic status and all described inequalities whereby low socioeconomic status groups were hit harder (Table 1). Most studies that found either no correlations or a correlation showing a greater impact on socioeconomically more affluent groups, were not peer-reviewed publications on preprint servers. Only one peer-reviewed study in the LitCovid database found a lower incidence of COVID-19 correlated to US regions with higher levels of unemployment [18].

Even though not all of the included studies reach the same conclusions, studies from the US and England with large numbers of cases show a clear picture of the degree of socioeconomic inequality in COVID-19. From England, Niedzwiedz et al. [66] report from the UK Biobank cohort study that people from socioeconomically strongly deprived regions have a 2.2 times higher risk of being tested positive for SARS-CoV-2. Price-Haywood et al. [40] report, based on a retrospective cohort study from the US with 3,481 patients who tested positive for SARS-CoV-2, that people from regions with a high proportion of low income earners have a higher risk of being admitted to hospital after developing COVID-19. For severe clinical courses, studies from England, Wales and Northern Ireland all indicate that among the 9,777 cases considered, patients from socioeconomically deprived regions were overrepresented in intensive care unit admissions [80]. For COVID-19 mortality, as well, the most conclusive findings on socioeconomic inequalities come from England and Wales. Williamson et al. [77] for example report from a large cohort study with 5,683 patients who had died after developing COVID-19 that people from the most deprived regions had more than twice the risk of dying from COVID-19 compared to people from the least deprived regions. Analyses of cause of death statistics from England and Wales confirm this finding: in its report from 12 June 2020, the national statistical office, after analysing 46,687 COVID-19-related deaths, finds that people from the most deprived regions had a roughly twice as high risk of dying from a SARS-CoV-2 infection than people from the least deprived regions [79].

While most studies present findings either from the US or the UK, only a few publications with results for European Union countries were found. A not peer-reviewed publication from the early stage of the epidemic in northern Italy with data that was collected up to 30 March 2020 found that the relative increase of incidence in 36 northern Italian provinces correlated positively with the regional employment rate, as well as regional population density and in-house density [62]. The authors concluded that this correlation was due to the greater mobility generally related to the variables, which, in the early stages of the pandemic, led to regional differences in incidence increases.

Up to the cut-off day for our literature review (15 June 2020), we found one empirical study for Germany. The not peer-reviewed publication by Plümper and Neumayer [48] analyses cumulative incidence rates for 401 German districts for two periods of time. The authors report a higher COVID-19 incidence for regions with higher incomes, higher education status, as well as a low proportion of recipients of social security benefits for the period up to 13 April 2020. For the second period analysed, from 14 April 2020 to 17 May 2020, the authors report that these correlations had reversed [48].

## 4. Discussion

The objective of this article was to provide an overview of the current knowledge status regarding the socioeconomic inequalities during COVID-19. Our scoping review showed that, in particular, studies from the US and the UK reported socioeconomic inequalities during the spread of the COVID-19 pandemic. Here, people from socioeconomically disadvantaged groups have a higher infection risk, are more frequently hospitalised and receive intensive care, and also have higher COVID-19 mortality rates than people from socioeconomically more privileged groups. Studies differed greatly regarding the applied socioeconomic indicators, and some also showed better outcomes for low socioeconomic status groups or no inequalities for certain indicators and outcomes. Two studies that showed no correlation or a correlation where higher socioeconomic status groups were hit harder [45, 62], used data from the early stages of the pandemic and therefore possibly missed shifts in correlations that occurred over time. Such a shift in patterns of regional socioeconomic inequalities during the pandemic is for example described by the only study from Germany included in the review [48] which also pointed to socioeconomic inequalities to the disadvantage of socially more deprived groups over the course of the pandemic. These results are confirmed by a first analysis of surveillance data with a regional index of socioeconomic deprivation at the district level (see Focus-article Socioeconomic inequalities in the risk of SARS-CoV-2 infection – First results from an analysis of surveillance data from Germany in this issue of the Journal of Health Monitoring). An additional study from Germany, which was published after 15 June 2020 and therefore not included by our scoping review, reported socioeconomic inequalities at the individual level [85]. Dragano et al. analysed AOK health insurance data from Rhineland/Hamburg and found an increased hospitalisation risk for people on unemployment benefits compared to those in work. The authors assess that this is mainly related to a higher prevalence of chronic diseases in this group, which could be risk factors for a more severe COVID-19 course. For the German language region, the Competence Network Public Health COVID-19 [86], a network of researchers from a number of expert associations, is an important initiative that collects results on socioeconomic inequalities in COVID-19 infections [13] and on people with particular risks and makes this information freely available [21].

In the international literature, in summary, more and more findings indicate that during more advanced stages of the pandemic, people with low socioeconomic status could be hit harder by COVID-19 than people with higher socioeconomic status. As the global pandemic remains a dynamic situation, the analysis of international evidence should be repeated. Hopefully there will be more findings from other European countries, which will then lead to a more complete picture of the social epidemiological patterns of COVID-19. However, as this literature review highlights, the majority of publications are currently ecological studies that only use regional level indicators. Only a few studies also provide correlations at the level of the individual. One reason for this is that most of the available COVID-19 data sources such as surveillance data or hospital data lack individual-level socioeconomic data in many countries. This urgently calls for increasing the collection of socioeconomic indicators at the level of the individual, for example in studies not only on the prevalence of antibody conversion following a SARS-CoV-2 infection, but also in routine data that provide insights into clinical disease courses, in order to be able to analyse socioeconomic inequalities at the individual level as well and might therefore reduce the danger of ecological fallacies [87].

This study has strengths and limitations. It is the first attempt to provide a comprehensive picture of the international knowledge status about the pandemic based on a literature search carried out in a reproducible manner which, as far as we know, has so far not been published in this format. Nonetheless, the sources were limited to the most important databases and preprint servers, and selection and data extraction were conducted by a single researcher to enable a timely compilation of results. This could have led to publications being missed and a selective inclusion of studies closer to the biomedical focus of the data sources being used, leaving out other more social science oriented studies. Publication bias too is a potential issue, leading to a skewed publication of results that show socioeconomic inequalities. A larger proportion of the literature included in this analysis also followed the primary objective of explaining differences between ethnic groups in the US and the UK and only provided information on socioeconomic inequalities as secondary findings. Not peer-reviewed studies from preprint servers were also included to reflect the most recent developments in the knowledge of the pandemic. A systematic evaluation of the scientific quality of these publications was not conducted, what may have led scientifically flawed studies to be included which could have led to a bias. Due to the fast moving nature of both the outbreak of COVID-19 and the scientific publications on the pandemic, this first systematic analysis of the current state of research should be seen as a snapshot that can create a better understanding of the current situation of the pandemic, but which requires a timely review.

## Conclusion

The results of this literature review indicate that for an acute viral respiratory disease such as COVID-19, risks of infection and a severe course may be distributed unequally across society. The international literature, particularly some specific studies from the US and the UK, reports considerable inequalities with people in low socioeconomic status groups being hit harder. These trends of socioeconomic inequality urgently require further monitoring. For Germany and other countries of the European Union in particular, there is hardly any analysis of potential socioeconomic inequalities in COVID-19 so far. To better understand the underlying pathways of the socioeconomic inequalities in COVID-19, future studies should include high-quality sociodemographic variables at the individual level and thereby reveal potential new approaches for targeted measures of infection protection and control.

## Key statements

- The international research literature points towards socioeconomic inequalities in COVID-19.
- Data from the US and the UK indicate greater risks for infection and severe courses of COVID-19 for socioeconomically less privileged populations.
- Analysing social epidemiological patterns in COVID-19 will be important to prevent an exacerbation of health inequalities.
- The collection of more high quality sociodemographic data is crucial to further investigate the correlations between socioeconomic status and COVID-19.

## Figures and Tables

**Table 1:** Overview of socioeconomic indicators and reported associations with SARS-CoV-2 infection risk, as well as hospitalisations and mortality with regard to COVID-19 in publications selected in the scoping review Source: Own table

A) Regional socioeconomic indicators			
Indicator	Study	Country	Result
Regional income	Price-Haywood et al. [40]	USA	higher hospitalisation risk correlates with lower income
	Azar et al. [41]	USA	higher hospitalisation risk correlates with lower income
	Mollalo et al. [42]	USA	higher incidence correlates with lower income
	Abedi et al. [43]	USA	higher incidence correlates with higher income^*^
	Chow et al. [44]	USA	higher incidence correlates with lower income
	Guha et al. [45]	USA	no increase in mortality for low income groups^*^
	Li et al. [46]	USA	higher incidence correlates with higher income^*^
	Mukherji [47]	USA	higher incidence correlates with higher income^*^
	Mukherji [47]	USA	higher mortality correlates with higher income^*^
	Pluemper & Neumayer [48]	Germany	higher incidence correlates with higher income (study point 1)^*^
	Pluemper & Neumayer [48]	Germany	higher incidence correlates with lower income (study point 2)
	Sy et al. [49]	USA	lower incidence correlates with higher income
	Takagi et al. [50]	USA	no correlation between prevalence and income^*^
	Vahidy et al. [51]	USA	lower incidence correlates with higher income
	Whittle & Diaz-Artiles [52]	USA	higher incidence correlates with lower income
Regional income inequality	Mollalo et al. [42]	USA	higher incidence correlates with greater inequality
	Mukherji [47]	USA	higher incidence correlates with greater inequality
	Mukherji [47]	USA	higher mortality correlates with greater inequality
Regional poverty rate	Ramirez & Lee [53]	USA	higher mortality correlates with greater poverty
	Wadhera et al. [54]	USA	higher mortality correlates with greater poverty
	Wadhera et al. [54]	USA	higher hospitalisation risk correlates with greater poverty
	Abedi et al. [43]	USA	higher mortality correlates with greater poverty
	Cyrus et al. [55]	USA	higher mortality correlates with greater poverty
	Federgruen & Naha [56]	USA	higher mortality correlates with greater poverty
	Fielding-Miller et al. [57]	USA	higher mortality correlates with greater poverty
	Guha et al. [45]	USA	no higher mortality with greater poverty^*^
	Li et al. [46]	USA	higher incidence correlates with higher poverty
	Rose et al. [58]	UK	higher mortality correlates with greater poverty
	Takagi et al. [59]	USA	higher prevalence correlates with greater poverty
	Takagi et al. [50]	USA	higher prevalence correlates with greater poverty
	Takagi et al. [50]	USA	no correlation between mortality and poverty^*^
	Chen & Krieger [60]	USA	higher incidence correlates with greater poverty
	Chen & Krieger [60]	USA	higher mortality correlates with greater poverty
	Chen et al. [61]	USA	higher mortality correlates with greater poverty
Regional unemployment rate	Ramirez & Lee [53]	USA	higher mortality correlates with high levels of unemployment
	Millett et al. [18]	USA	lower incidence correlates with high levels of unemployment^*^
	Mukherji [47]	USA	lower mortality correlates with high levels of unemployment^*^
	Takagi et al. [59]	USA	higher prevalence correlates with high levels of unemployment
	Pluemper & Neumayer [48]	Germany	lower incidence correlates with high levels of unemployment (study point 1)^*^
	Pluemper & Neumayer [48]	Germany	higher incidence correlates with high levels of unemployment (study point 2)
Regional employment rate	Buja et al. [62]	Italy	higher incidence correlates with high levels of employment^*^
Regional education	Wadhera et al. [54]	USA	higher hospitalization risk correlates with low education
	Wadhera et al. [54]	USA	higher mortality correlates with low education
	Abedi et al. [43]	USA	higher incidence correlates with high education^*^
	Maroko et al. [63]	USA	higher incidence correlates with low education
	Takagi et al. [59]	USA	lower prevalence correlates with high education
	Xie & Li [64]	USA	higher incidence correlates with low education
	Pluemper & Neumayer [48]	Germany	higher incidence correlates with high education (study point 1)^*^
	Pluemper & Neumayer [48]	Germany	higher incidence correlates with low education (study point 2)
Regional deprivation indices	Kim & Bostwick [65]	USA	higher mortality correlates with high deprivation levels education
	Niedzwiedz et al. [66]	UK	higher risk of hospitalisation correlates with high deprivation levels
	Niedzwiedz et al. [67]	UK	higher incidence correlates with high deprivation levels
	Lassale et al. [68]	UK	higher risk of hospitalisation correlates with high deprivation levels
	Apea et al. [69]	UK	no correlation between mortality and deprivation^*^
	Ho et al. [70]	UK	higher incidence correlates with high levels of deprivation
	Khawaja et al. [71]	UK	higher incidence correlates with high levels of deprivation
	Liu et al. [72]	UK	higher incidence correlates with high levels of deprivation
	Nayak et al. [73]	USA	higher case fatality rate correlates with high levels of deprivation
	Nazroo et al. [74]	England and Wales	higher mortality correlates with high levels of deprivation
	Patel et al. [75]	England	higher risk of hospitalisation correlates with high deprivation levels
	Prats-Uribe et al. [76]	England	higher risk of hospitalisation correlates with high deprivation levels
	Raisi-Estabragh et al. [19]	England	higher incidence correlates with high levels of deprivation
	Williamson et al. [77]	England	higher mortality correlates with high levels of deprivation
	Public Health England [78]	England and Wales	higher incidence and mortality correlates with high levels of deprivation
	Office for National Statistics [79]	England and Wales	higher mortality correlates with high levels of deprivation
	Intensive Care National Audit & Research Centre [80]	England, Wales, North. Ireland	higher rates of hospitalisation in intensive care correlates with higher levels of deprivation
Regional insurance status	Millett et al. [18]	USA	higher incidence correlates with higher number of uninsured
	Fielding-Miller et al. [57]	USA	lower mortality correlates with high proportion of uninsured^*^
	Takagi et al. [59]	USA	lower prevalence with higher proportion of privately insured patients
Regional living conditions	Millett et al. [18]	USA	higher incidence correlates with high proportion of people in crowded housing
	Ahmad et al. [81]	USA	higher incidence correlates with high proportion living in poor housing conditions
	Ahmad et al. [81]	USA	higher mortality correlates with high proportion of people in poor housing conditions
	Khanijahania [82]	USA	higher incidence for people who spend higher fractions of their income on housing
	Xie & Li [64]	USA	higher incidence for people who spend higher fractions of their income on housing
**B) Individual socioeconomic indicators**			
Income	Okoh et al. [83]	USA	higher risk of hospitalisation correlates with low income
	Okoh et al. [83]	USA	higher risk of hospitalisation correlates with low income
	Lassale et al. [68]	England	higher risk of hospitalisation correlates with low income
	Patel et al. [75]	England	higher risk of hospitalisation correlates with low income
Education	Lassale et al. [68]	England	higher risk of hospitalisation correlates with low education
	Niedzwiedz et al. [66]	England	higher incidence correlates with low education
Profession	Lassale et al. [68]	England	higher risk of hospitalisation correlates with blue collar jobs
Insurance status	Price-Haywood et al. [40]	USA	higher risk of hospitalisation for healthcare patients
	Azar et al. [41]	USA	higher risk of hospitalisation for healthcare patients
Housing	Raisi-Estabragh et al. [19]	England	higher incidence correlates with crowded living conditions
Profession	Public Health England [78]	England and Wales	higher incidence and mortality for certain professions
	Office for National Statistics [84]	England and Wales	higher mortality for unskilled labour and certain professions

^*^ socioeconomic inequalities with high status groups being more affected or no correlation

Colour codes: blue = peer-reviewed publication (LitCovid), grey = not peer-reviewed publication, white = official reports
